# Development of a Stereo Vision Measurement System for a 3D Three-Axial Pneumatic Parallel Mechanism Robot Arm

**DOI:** 10.3390/s110202257

**Published:** 2011-02-21

**Authors:** Mao-Hsiung Chiang, Hao-Ting Lin, Chien-Lun Hou

**Affiliations:** Department of Engineering Science and Ocean Engineering, National Taiwan University, No.1, Sec. 4, Roosevelt Rd., Taipei 10617, Taiwan; E-Mails: d97525009@ntu.edu.tw (H.-T.L.); cl_hou@yahoo.com.tw (C.-L.H.)

**Keywords:** stereo vision, 3D reconstruction, passive perception, circle detection, image rectification, parallel mechanism robot arm

## Abstract

In this paper, a stereo vision 3D position measurement system for a three-axial pneumatic parallel mechanism robot arm is presented. The stereo vision 3D position measurement system aims to measure the 3D trajectories of the end-effector of the robot arm. To track the end-effector of the robot arm, the circle detection algorithm is used to detect the desired target and the SAD algorithm is used to track the moving target and to search the corresponding target location along the conjugate epipolar line in the stereo pair. After camera calibration, both intrinsic and extrinsic parameters of the stereo rig can be obtained, so images can be rectified according to the camera parameters. Thus, through the epipolar rectification, the stereo matching process is reduced to a horizontal search along the conjugate epipolar line. Finally, 3D trajectories of the end-effector are computed by stereo triangulation. The experimental results show that the stereo vision 3D position measurement system proposed in this paper can successfully track and measure the fifth-order polynomial trajectory and sinusoidal trajectory of the end-effector of the three- axial pneumatic parallel mechanism robot arm.

## 1. Introduction

Many manufacturing processes use robots to perform various tasks, include welding, assembling, pick and place, and defect inspection. All these tasks require knowledge of the relative location between the robot’s end-effector and the desired target. The best-known technique to determine three-dimensional location information is based on stereo vision. Stereo vision systems often consist of two or multiple imaging devices along with a PC or other microprocessors. Due to the advantages of cost, easy maintenance, reliability, and non-contact measurement, stereo vision has become a popular research topic and been applied in industrial automation, autonomous vehicles, augmented reality, medical, and transportation [1–4].

A three-axial pneumatic parallel mechanism robot arm developed by NTU-AFPC Lab [5] was the test rig in this study. Its end-effector is able to follow the desired trajectories by controlling the positions of three rod-less pneumatic cylinders using nonlinear servo control. However, the kinematic model of the test rig has many different solutions so the real trajectories of the end-effector cannot be known only by the measured position of the three pneumatic actuators. To solve this problem, a common method is to use angular sensors for measuring angular displacements of each joint, and the trajectory of the end-effector can be calculated and estimated by the robot forward kinematics. Compared the joint angle measuring method with the stereo vision method, the stereo vision method has the advantages of involving non-contact sensing and providing direct measurements. Besides, it’s very difficult to fit angular sensors at the joints of parallel mechanism of the robot due to the restrictions of the mechanism used in this study.

To reconstruct a 3D space by stereo images using binocular cues, the disparities of the corresponding points in stereo pairs have to be known. Therefore, solving the stereo correspondence problem has been the most important stage on the 3D reconstruction. Some published studies [6–9] have attempted to solve the stereo correspondence problem. The most popular and well-known method is to use epipolar constraints [10] to reduce the stereo matching region from an area to a straight line.

For an uncalibrated stereo rig (*i.e.*, both intrinsic and extrinsic parameters are unknown) [11–13], the fundamental matrix *F* often needs to be computed to express epipolar constraints on uncalibrated stereo pairs. In contrast, a calibrated stereo rig with known intrinsic and extrinsic parameters can use so-called epipolar rectification [14,15] to transform each corresponding stereo pair for making the epipolar lines parallel and at the same horizontal rows, which greatly reduces the stereo matching region to a horizontal row. In this paper, the epipolar rectification algorithm [15] is adapted, which is a compact and clear stereo rectification algorithm and can provide MATLAB code for research reference. Since the algorithm assumes that the stereo rig is calibrated, camera calibration needs to be performed first.

The camera calibration procedure in this paper was accomplished using the Camera Calibration Toolbox of MATLAB [16] developed by Bouquet. Bouquet’s main initialization phase of camera calibration inspires by Zhang [17] that uses a chessboard as calibration pattern to obtain both intrinsic and extrinsic camera parameters, and Bouquet’s intrinsic camera model inspired by Heikkilä and Silvén [18] which includes two extra distortion coefficients of Zhang’s intrinsic camera model to get more precise stereo rectification. After stereo rectification is done, the correspondence problem of desired target is solved.

The Circle Hough Transform (CHT) [19] is one of the best known methods which aims to detect lines or circles in an image. The algorithm transforms each edge point in edge map to parameter space and plots histogram of parameter space in an accumulator space as output image in which the highest-frequency accumulator cell (*i.e.*, pixel with the highest gray value) is the outcome. If circle radii are unknown, the parameter space is a three-dimensional space, and that requires a large amount of computing power for the algorithm, which is the major drawback for real-time application. Kimme *et al*. [20] first suggested that using edge direction can reduce the computational requirements of the CHT to only an arc to be plotted in the accumulator space. Minor and Sklansky [21], and Faez *et al.* [22] then proposed that plotting a line in the edge direction has the advantage of reducing parameter space to a two dimensional space. Scaramuzza *et al*. [23] developed a new algorithm which rejects non-arc segments (e.g., isolated points, noises, angular points, and straight segments) and plots lines in the direction of arc concavity. The algorithm gives more precise approximation for circle location.

There are two major procedures in stereo vision tracking, including motion tracking and stereo matching [24]. The location of the desired target in the reference image (e.g., the left image) is tracked by using the motion tracking algorithm, and the stereo matching algorithm is then matching the correspondence location of the desired target in the other image (e.g., the right image).

Motion tracking involves two types of algorithms: feature-based tracking algorithm [25,26] and region-based tracking algorithm [27–29]. The feature-based tracking algorithm tracks partial features of the target. The canny edge detector [30] is often used for extracting edge features of the target, and point feature of the target’s corner is extracted by the SUSAN corner detector [31]. Region-based tracking algorithm uses the template/block determined by user selection or image recognition to track the target. Once the template/block is decided, the algorithm starts to compute the correlation between the template/block and the designated region in the current frame. The most common used correlation criteria are the sum of absolute differences (SAD) and the sum of squared differences (SSD). References [28,29] suggested the template update strategies that solve the “drifting” problem caused by environmental influence (e.g., light conditions or object occlusion) during motion tracking.

The developed stereo matching methods can roughly be divided into two categories: local methods and global methods [32]. Although global methods, such as those using dynamic programming [33], can be less sensitive to local ambiguous regions (e.g., occlusion regions or regions with uniform texture in an image) than those using the local method, the global methods require more computing cost [34]. Block matching [35] is the best known method among the local methods because of its efficiency and simplicity in implementation. In the block matching, the reference block determined in moving tracking is used to search stereo corresponding by using matching criteria such as SSD or SAD. Once the stereo matching is made, each corresponding locations of the target in the stereo images are found, that is, the disparity of the target’s location is known. Therefore, the depth information of the target can be calculated by triangulation.

This paper aims to develop a stereo vision system that is considered as a sensor to measure 3D space trajectories of the end-effector of the three-axial pneumatic parallel mechanism robot arm in real-time. Thus, the real-time stereo tracking is required to ensure that the stereo measurement process and the end-effector’s motion are as synchronized as much as possible. The Multimedia Extension (MMX) technology [6] is utilized in this paper to minimize the computational cost of the stereo tracking process. In addition, the stereo depth estimation will be calibrated by the linear encoder measuring results on a straight-line moving pneumatic cylinder. Therefore, the correctness of stereo vision system can be known. For that, a test rig is set up for realizing the developed strategies of the stereo vision which is used to measure the end-effector of the three-axial pneumatic parallel mechanism robot arm.

## 2. System Setup

**Figure 1.**
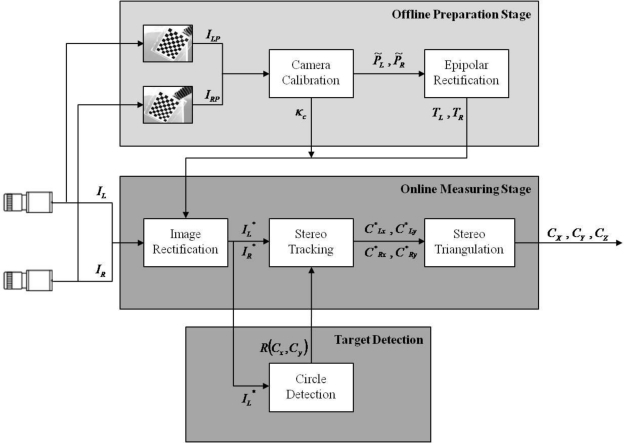
System overview.

The system setup combines the stereo vision measuring method with the three-axial pneumatic parallel mechanism robot arm for measuring the 3D trajectories of the end-effector. Based on the structure design, the position of the end-effector of the three-axial pneumatic parallel mechanism robot arm is very difficult to measure directly in the practical experiment. It can be calculated via the position sensors of the three linear actuators by means of the kinematics. However, there are many different solutions for the kinematics of the end-effector of the three-axial pneumatic parallel mechanism robot arm, so the accurate xyz coordinates of the end-effector are difficult to solve. Thus, this paper proposes the stereo vision measuring method to measure the absolute xyz coordinates of the end-effector after the image coordinate calibration. In this paper, the stereo vision measurement system can be divided into two parts: the offline preparation stage and the online measuring stage, as shown in Figure 1.

The offline preparation stage of the system includes camera calibration and calculation of transformation matrices for epipolar rectification, and a series of calibration patterns must be taken for this stage. After each camera of the stereo rig is calibrated independently, both projection matrices and radial distortion coefficients of the left and right cameras are used to compute the transformation matrices and to rectify the distorted images.

In the online measuring stage, the transformation matrices and the radial distortion coefficients calculated at the offline preparation part are imported for the image rectification. In the first place, the desired target is detected in the rectified left image by means of circle detection, and the reference block for stereo tracking is defined.

**Figure 2.**
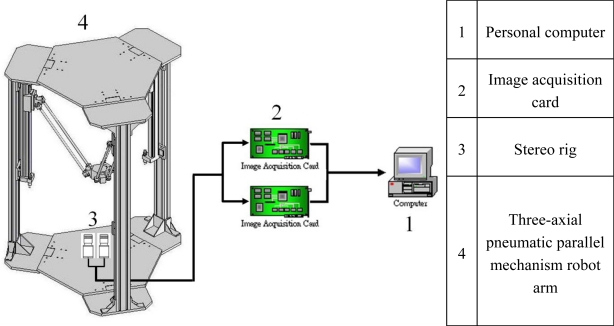
Stereo vision measurement system scheme.

Once all requirements mentioned above are computed, the real-time 3D measurement can be executed. The target locations in both left and right image are tracked by stereo tracking so as to compute the estimated 3D world coordinates of the target through stereo triangulation. Figure 2 shows the system scheme of the stereo vision measurement system.

**Figure 3.**
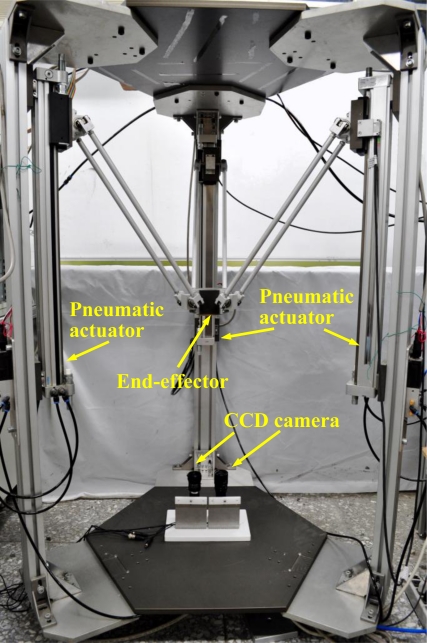
The layout of the test rig.

The stereo rig in this study, as shown in Figure 3, is composed of two identical CCD cameras which are equipped with camera lenses, and the baseline distance is approximately 7 cm. Figure 3 also shows the three-axial pneumatic parallel mechanism robot arm developed by the NTU-AFPC lab [5]. The end-effector is the desired target of the stereo vision measurement system.

Based on image quality and anti-noise performance, the CCD image sensor is better than the CMOS image sensor, thus, the CCD camera is selected in this paper [36]. A camera manufactured by TOSHIBA TELI, model CS8550Di, which supports progressive area scan and interlaced area scan, is utilized in this paper For the real-time application aspect, the progressive area scan is used in this paper. The detailed specificationd of the camera are shown in Table 1.

An analog CCD camera has analog signals, so it needs an image acquisition device to digitalize the analog signals for further processing or analyzing on a PC or other processors. The image acquisition card developed by National Instruments, model IMAQ PCI-1410 is chosen in this paper. It has 16 MB of onboard SDRAM used to temporarily store the image being transferred to the PCI bus, and three independent onboard DMA (Direct memory access) controllers for improving its performance. The intensity resolution can reach 10 bit/pixel, and 8-bit pixel format is supported on software programming. Table 1 shows its detailed specifications.

## 3. Image Rectification

Given a pair of stereo images, the problem of finding pixels or objects in one image which can be identified as the same pixels or objects in another image is called the correspondence problem. Solving the correspondence problem is difficult, due to problems such as object occlusion, lens distortion, aperture problem, and homogeneous regions in the stereo pair [39]. These problems make the correspondence problem difficult and complex. To make it easier, the image rectification is introduced.

Rectifying stereo images involves finding a transformation for each image plane such that pairs of conjugate epipolar lines become collinear and parallel to one of the image horizontal axes. Because the search of corresponding points becomes only along the horizontal lines of rectified images, the stereo rectification makes the correspondence problem easier. In the following sections, the stereo-pair image rectification methods applied in this paper will be introduced.

### 3.1. Camera Model

**Figure 4.**
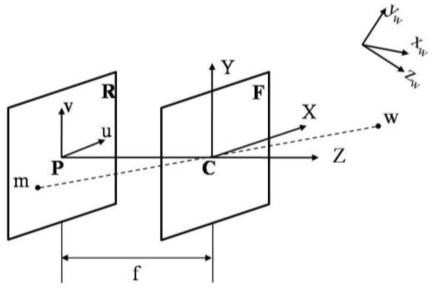
The pinhole camera model.

To rectify the stereo images, the knowledge of camera model and its parameters are important. Figure 4 depicts the pinhole camera model, which is the simplest camera model that describes the mathematical relationship between the 3D world coordinates and the image plane coordinates.

*R* is the image plane (or retinal plane) centered on the principle point *P*; *F* is the focal plane centered on the optical center *C*. Both planes are parallel to the focal length *f*. The straight line passing through the principle point *P* and the optical center *C* is called the optical axis.

A three-dimensional point $\tilde{\text{w}}={\left[\begin{array}{cccc} x & y & z & 1 \end{array}\right]}^{T}$ is defined to be the homogeneous coordinates in the world reference frame $\left\{\begin{array}{ccc} x_{w} & y_{w} & z_{w} \end{array}\right\}$ (fixed arbitrarily) and $\tilde{\text{m}}={\left[\begin{array}{ccc} u & v & s \end{array}\right]}^{T}$ is defined to be the homogeneous coordinates in the image frame {*u v*} (pixels) where *s* is the scale factor. Assume that a homogeneous transformation matrix **P̃** exists and is given by:

(1) $$\tilde{\text{P}}=\left[\begin{array}{c} q_{1}^{T}\\ q_{2}^{T}\\ q_{3}^{T} \end{array}|\begin{array}{c} q_{14}\\ q_{24}\\ q_{34} \end{array}\right]=\left[Q|\tilde{\text{q}}\right]$$

which represents the mapping relationship between the world reference frame and the image frame; the relationship can be formulated as:

(2) $$\tilde{\text{m}}=\tilde{\text{P}}\tilde{\text{w}}$$

In Equation (1), the homogeneous transformation matrix **P̃** is also called the perspective projection matrix (PPM), which can be considered as the combination of transformations: the extrinsic parameters **T***_e_* and the intrinsic parameters **T***_i_*. Therefore, the homogeneous coordinates in the image frame **m̃** can be written as:

(3) $$\tilde{\text{m}}=\tilde{\text{P}}\tilde{\text{w}}=T_{i}T_{e}\tilde{\text{w}},\;\left[\begin{array}{c} u\\ v\\ s \end{array}\right]=T_{i}T_{e}\left[\begin{array}{c} x_{w}\\ y_{w}\\ z_{w}\\ 1 \end{array}\right]$$

The extrinsic parameters **T***_e_* define the position and the orientation of the camera reference frame with respect to the world reference frame by a rotation **R** and a translation **t**:

(4) $$\left[\begin{array}{c} X\\ Y\\ Z \end{array}\right]=T_{e}\;\left[\begin{array}{c} x_{w}\\ y_{w}\\ z_{w}\\ 1 \end{array}\right],\;\;T_{e}=\left[R|\;t\right]=\left[\begin{array}{cccc} r_{11} & r_{12} & r_{13} & t_{1}\\ r_{21} & r_{22} & r_{23} & t_{2}\\ r_{31} & r_{32} & r_{33} & t_{3} \end{array}\right]$$

The intrinsic parameters **T***_i_* are the optical characteristics and the internal geometric of the camera, which define the pixel coordinates of image frame with respect to the coordinates in the camera reference frame:

(5) $$\left[\begin{array}{c} u\\ v\\ s \end{array}\right]=T_{i}\;\left[\begin{array}{c} X\\ Y\\ Z \end{array}\right],\;\;T_{i}=\left[\begin{array}{ccc} \alpha & \gamma & u_{o}\\ 0 & \beta & v_{o}\\ 0 & 0 & 1 \end{array}\right]$$

In Equation (5), where *α* = *f/k*_0_ and *β* = *f/k*_1_ are the focal lengths in horizontal and vertical pixels respectively (*f* is the focal length in millimeter, and (*k*_0_, *k*_1_) are the pixel size in millimeter), (*u*_0_, *v*_0_) are the coordinates of the principle point, and *γ* is the skew factor that models non-orthogonal *u*–*v* axes. Since (*u*, *v*, *s*) is homogeneous, the pixel coordinates *u*′ and *v*′ can be retrieved by dividing the scale factor *s*.

The camera model derived above is based on the simple pinhole camera model, which doesn’t take the lens distortion into consideration. To correct the radial distortion image, the lens distortion model implemented by Devernay *et al.* [37] is included in the camera model. As shown in Equations (6) and (7), an infinite polynomial series is used to model the radial distortion, in which, *κ*_1_ and *κ*_2_ are the first and second order radial distortion parameters, and *r_d_* is the distorted radius. Note that *x_d_* and *y_d_* are the distorted camera coordinates; *x_d_* and *y_u_* are the undistorted camera coordinates:

(6) $$x_{u}=x_{d}\;\left(1+\kappa_{1}{r_{d}}^{2}+\kappa_{2}{r_{d}}^{4}+\cdots \right)$$

(7) $$y_{u}=y_{d}\;\left(1+\kappa_{1}{r_{d}}^{2}+\kappa_{2}{r_{d}}^{4}+\cdots \right)$$

(8) $$r_{d}=\sqrt{{x_{d}}^{2}+{y_{d}}^{2}}$$

By eliminating higher-order terms of Equations (6) and (7), which can be written as Equations (9) and (10), respectively:

(9) $$x_{u}=x_{d}\;\left(1+\kappa_{1}{r_{d}}^{2}+\kappa_{2}{r_{d}}^{4}\right)$$

(10) $$y_{u}=y_{d}\;\left(1+\kappa_{1}{r_{d}}^{2}+\kappa_{2}{r_{d}}^{4}\right)$$

If camera has been calibrated, that is, the intrinsic parameters are known, so the radial distortion parameters and the distorted camera coordinates can be computed by Equation (11); the radial distortion correction can then be achieved through Equations (12) and (13):

(11) $$\left[\begin{array}{c} x_{d}\\ y_{d}\\ 1 \end{array}\right]=T_{i}^{-1}\left[\begin{array}{c} u_{d}\\ v_{d}\\ 1 \end{array}\right]$$

(12) $$u_{u}=u_{d}\;\left(u_{d}-u_{0}\right)\left(\kappa_{1}{r_{d}}^{2}+\kappa_{2}{r_{d}}^{4}\right)$$

(13) $$v_{u}=v_{d}\;\left(v_{d}-v_{0}\right)\left(\kappa_{1}{r_{d}}^{2}+\kappa_{2}{r_{d}}^{4}\right)$$

### 3.2. Camera Calibration

Camera calibration is a process to find the intrinsic parameters and the extrinsic parameters of a camera. The knowledge of these parameters is essential for the stereo rectification. The Camera Calibration Toolbox of MATLAB is adopted as the camera calibration tool in this paper to find parameters of the stereo rig.

**Figure 5.**
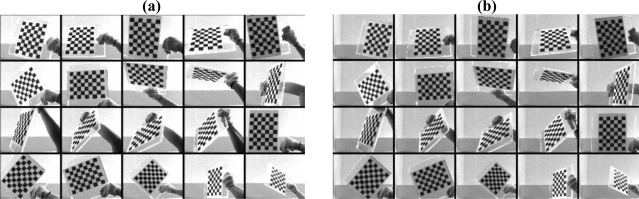
The calibration patterns. **(a)** Left images; **(b)** Right images.

In order to obtain precise parameters of camera, the calibration pattern needs to take at least 5 and up to 20 pictures from different distances and angles simultaneously. As shown in Figure 5, the pictures of the chess board are taken by the left and the right camera respectively.

After the stereo images of the calibration patterns are achieved, the camera calibration tool is able to compute the intrinsic parameters and the extrinsic parameters of each camera.

### 3.3. Epipolar Geometry

**Figure 6.**
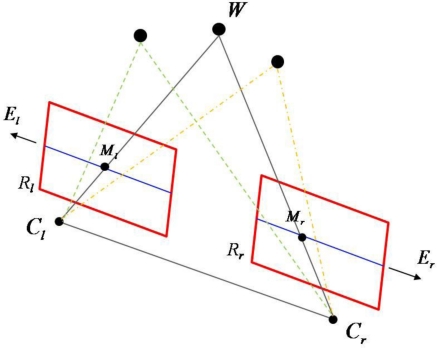
The standard setting of cameras.

As shown in Figure 6, it is interesting to note that when the baseline ( $\bar{C_{r}C_{l}}$) is contained in both focal planes, that is, both image planes (*R_l_* and *R_r_*) are parallel to the baseline, the epipoles (*E_r_* and *E_l_*) are at infinity and the epipolar lines, denoted by the blue lines on two image planes, are all horizontal.

In this special case, also called the standard setting, the epipolar lines corresponding to the same horizontal rows with the same *y* coordinate in both images and point correspondences are searched over these rows, and that simplifies the computation of stereo correspondences. The imaged points of three arbitrary 3D points are all on the same horizontal epipolar lines.

### 3.4. Epipolar Rectification

As mentioned, the standard setting has a great advantage of reducing the computation of stereo correspondences, but it cannot be obtained by real cameras. However, if the cameras’ calibration parameters are known, this problem could be overcome through the Epipolar Rectification. In this paper, the rectification algorithm presented by Fusiello *et al.* [15] is adopted.

The stereo rig can be calibrated by Bouguet’s stereo calibration tool, that is, the intrinsic and extrinsic parameters of both left and right cameras are known. Therefore, from Equation (1), the PPM of the left camera **P̃***_ol_* and the PPM of the right camera **P̃***_or_* can be written as:

(14) $${\tilde{\text{P}}}_{\mathrm{ol}}=\left[Q_{\mathrm{ol}}|\;q_{\mathrm{ol}}\right],\;\;{\tilde{\text{P}}}_{\mathrm{or}}=\left[Q_{\mathrm{or}}|\;q_{\mathrm{or}}\right]$$

and the coordinates of the left optical center **c***_l_* and the right optical center **c***_r_* can be determined as:

(15) $$c_{l}=-{Q_{\mathrm{ol}}}^{-1}q_{\mathrm{ol}},\;\;\;c_{r}=-{Q_{\mathrm{or}}}^{-1}q_{\mathrm{or}}$$

Define a pair of new PPMs **P̃***_nl_* and **P̃***_nr_* as:

(16) $${\tilde{\text{P}}}_{\mathrm{nl}}=T_{\mathrm{ni}}\left[R|-Rc_{l}\right],\;\;{\tilde{\text{P}}}_{\mathrm{nr}}=T_{\mathrm{ni}}\left[R|-Rc_{r}\right],$$

where the new intrinsic parameters **T***_ni_* is the same for both new PPMs, and can be chosen arbitrarily; the optical centers **c***_l_* and **c***_r_* are computed in Equation (15) of the old PPMs. The rotation matrix **R** is the same for both new PPMs, which will be specified by means of its row vectors:

(17) $$R=\left[\begin{array}{c} {\hat{\text{x}}}^{T}\\ {\hat{\text{y}}}^{T}\\ {\hat{\text{z}}}^{T} \end{array}\right]$$

The row vectors of **R** are the X, Y, and Z axes, respectively, of the camera reference frame, expressed in the world coordinates.

According to Fusiello’s algorithm, the row vectors of **R** can be calculated as:

1. The new X axis is parallel to the baseline:
  $$\hat{\text{x}}=\left(c_{l}-c_{r}\right)/\left\|c_{l}-c_{r}\right\|$$
2. The new Y axis is orthogonal to X (mandatory) and to **k̂**:
  $$\hat{\text{y}}=\hat{\text{k}}\wedge \hat{\text{x}}$$
3. The new Z axis is orthogonal to XY (mandatory):
  $$\hat{\text{z}}=\hat{\text{x}}\wedge \hat{\text{y}}$$

In the calculation of the new Y axis, the vector **k̂** is an arbitrary unit vector which makes the new Y axis orthogonal to the new X axis. In order to make the new Y axis orthogonal to both the new X axis and the old Z axis, **k̂** is set to be the unit vector of the old Z axis.

To rectify the left and right images, the mapping relationships between the old PPMs and the new PPMs of the left and right images need to be known. Let us consider the left image as the example here.

For a 3D point **w** appears on the left and right cameras, the old left perspective projection **m̃***_ol_* and the new left perspective projection **m̃***_nl_* can be expressed as:

(18) $${\tilde{\text{m}}}_{\mathrm{ol}}≅{\tilde{\text{P}}}_{\mathrm{ol}}\tilde{\text{w}},\;{\tilde{\text{m}}}_{\mathrm{nl}}≅{\tilde{\text{P}}}_{\mathrm{nl}}\tilde{\text{w}}$$

Because the optical center is not affected by rectification, Equation (18) can be expressed in its parametric form as:

(19) $$\{\begin{array}{ll} w=c_{l}+\lambda_{o}Q_{\mathrm{ol}}^{-1}{\tilde{\text{m}}}_{\mathrm{ol}} & \lambda_{o}\in R\\ w=c_{l}+\lambda_{n}Q_{\mathrm{nl}}^{-1}{\tilde{\text{m}}}_{\mathrm{nl}} & \lambda_{n}\in R \end{array}$$

Therefore:

(20) $${\tilde{\text{m}}}_{\mathrm{nl}}=\frac{\lambda_{o}}{\lambda_{n}}Q_{\mathrm{nl}}Q_{\mathrm{ol}}^{-1}{\tilde{\text{m}}}_{\mathrm{ol}}=\lambda Q_{\mathrm{nl}}Q_{\mathrm{ol}}^{-1}{\tilde{\text{m}}}_{\mathrm{ol}}\;\;\;\;\;\lambda \in R$$

from Equation (20), the transformation mapping the old left image onto the new left image is derived as:

(21) $$T_{l}=Q_{\mathrm{nl}}Q_{\mathrm{ol}}^{-1}$$

and the transformation of the right image applies the same result of the left image:

(22) $$T_{r}=Q_{\mathrm{nr}}Q_{\mathrm{or}}^{-1}$$

**Figure 7.**
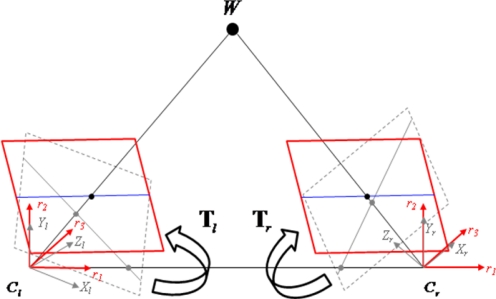
The epipolar rectification.

Now the rectification transformations **T***_l_* and **T***_r_* have been derived, which can be applied to the original left and right image, respectively, to get the rectified images. Figure 7 illustrates the above rectification transformation. Note that the bilinear interpolation is applied to interpolate the non-integer positions of the rectified images to the corresponding pixel coordinates of the original images.

**Figure 8.**
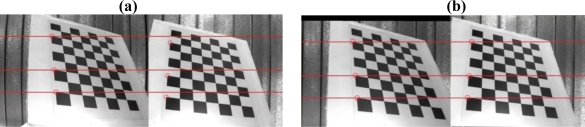
Image rectification result. A stereo pair: **(a)** before being rectified **(b)** after epipolar rectification.

The image rectification includes the radial distortion correction [37] and the epipolar rectification [15] in this paper. Since both need to have the knowledge of camera parameters, Bouguet’s camera calibration toolbox of MATLAB [16] is used. The radial distortion correction and the epipolar rectification can be carried out, and the results are shown in Figure 8. Table 2 shows the intrinsic and extrinsic parameters of the stereo rig.

## 4. Target Detection

**Figure 9.**
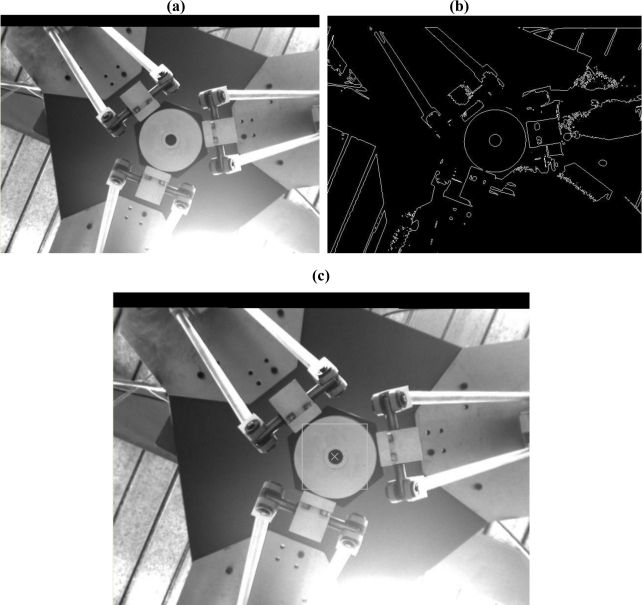
The target detection results: **(a)** The end-effector of robot arm, **(b)** After Laplacian operator of Figure 5(a), **(c)** Target detected and marked in a white square.

When detecting a circular object in a 3D space the circle radius is unknown. Although the circle radius can be known by pixel measurement in an image, it either needs to be re-measured when the circle radius changes, or the target has to be set in the vertical plane to maintain the same circle radius. It’s inconvenient and loses the generality. Hence, the ‘Pixel-to-Pixel’ circle detection algorithm developed by D. Scaramuzza *et al.* [23] is adopted in this paper. The experimental results of the target detection are shown in Figure 9. Note that the target detection algorithm is applied on the rectified left image. Figure 9(a) is the target required to be detected; Figure 9(b) is the image applied by the Laplacian edge detection to Figure 9(a); Figure 9(c) shows the result of target detection.

## 5. Stereo Tracking

The blocking matching is one of the best known methods for motion tracking and stereo matching due to its ease of implementation and less computational effort. The Sum of Square Difference (SSD) and the Sum of Absolute Difference (SAD) are the commonly used matching criteria for block matching. Because the SSD squares the intensity differences, it requires heavier computational burden than the SAD during the matching process. For the purpose of real-time stereo tracking, the SAD based stereo tracking is utilized:

(23) $$\begin{array}{l} \mathrm{SAD}\left(x,\;y\right)=\sum_{j=0}^{2n}\;\;\sum_{i=0}^{2n}\;\;\left|f_{k}\left(x+i,\;y+i\right)-R\left(i,\;j\right)\right|\\ \left(x_{b},\;y_{b}\right)=\underset{\left(x,\;y\right)\in W_{k}}{\text{min}}\left(\mathrm{SAD}\left(x,\;y\right)\right) \end{array}$$

Equation (23) expresses the SAD matching criterion, where *f_k_* is an image from the *k* frame; *R* is selected the reference block; (2*n* + 1) × (2*n* + 1) is the size of the reference block.

**Figure 10.**
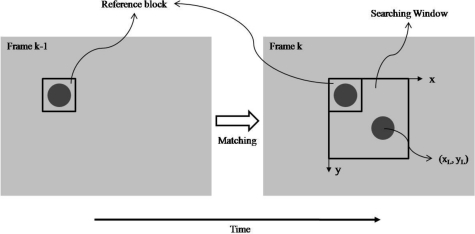
The block matching process.

After the circle detection determined the location of the object at the previous frame, the reference block of size (2*n* + 1) × (2*n* + 1) centered on this location is created and stored in memory to search the best match (*i.e.*, the SAD score has the minimum value) at the current frame in the searching window. Once the best match has been found, the current location of the object (*x_L_*,*y_L_*) in the left image can be tracked; in addition, the reference block is replaced by the best match to adapt the searching on the next frame. Figure 10 illustrates the block matching process mentioned above.

Assume that the left and right images have been rectified, so the reference block only searches horizontally along the epipolar line of the right image. The searching criterion is the same as the moving tracking; that is, the best match is determined at the location where the SAD score is the minimum.

**Figure 11.**
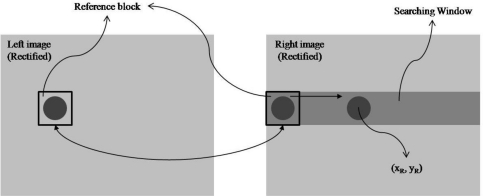
The stereo matching.

When the best match has been found, the corresponding location of the object in the right image can be obtained. Figure 11 shows the stereo matching process. In Equation (24), the SAD score is used to estimate the similarity between the reference block *R_l_* of the left image and right image *I_r_*. The search for the best match is done consecutively along all possible candidates within the allowable disparity range *d*_min_ ≤ *d_c_* ≤ *d*_max_ :

(24) $$\mathrm{SAD}\left(x,\;y,\;d_{c}\right)=\sum_{j=0}^{2n}\;\;\sum_{i=0}^{2n}\;\;\left|R_{l}\left(x+i,\;y+i\right)-I_{r}\left(x+i+d_{c},\;y+j\right)\right|$$

**Figure 12.**
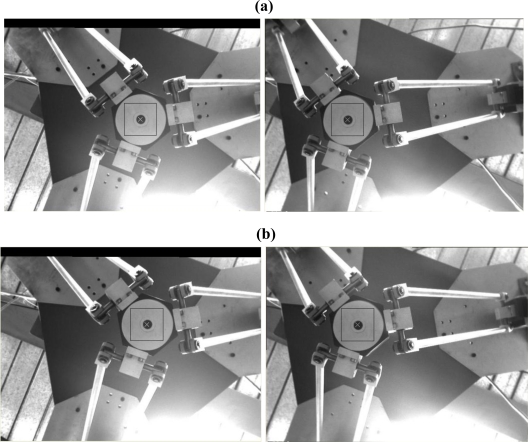

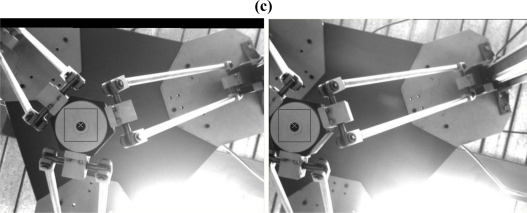
Left and right image stereo tracking results when the target at an arbitrary position A **(a)**, position B **(b)** and position C **(c)**.

Figure 12(a,c) shows the stereo tracking results at three arbitrary positions of the target, namely, positions A, B, and C. The reference block is defined as a 25 × 25 size rectangular block, and the size of searching window is 50 × 50 in the left image. For stereo matching on the right image, the row size of the horizontal scan-line is 20 ≤ *x_r_* ≤ 360. Once the first match is found, the row size of the horizontal scan-line becomes $\left(x_{r}^{*}-37\right)\leq x_{r}\leq \left(x_{r}^{*}+13\right)$, where $x_{r}^{*}$ is the *x*-coordinates of the first match.

## 6. Measurement Correction

**Figure 13.**
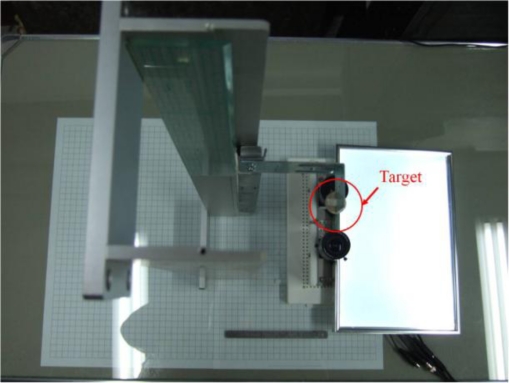
Measurement calibration method.

There are three major types of errors in the correlation-based stereo system, such as foreshortening error, misalignment error, and systematic error [38]. In order to correct the incorrect measuring results caused by these error sources, the stereo system needs to be calibrated. Figure 13 shows the measurement calibration method used in this thesis. A one-centimeter grid paper and a custom-made mechanism are used to calibrate the measurement results.

Table 3 is the error table of depth measurement, which shows the depth measurement error on each corresponding *X_W_* coordinate. Note that the blank in the table indicates that the target goes out the view field of the stereo rig.

**Figure 14.**
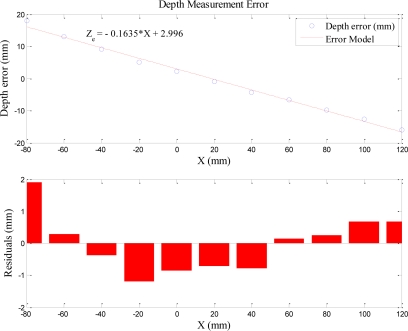
Linear depth measurement error model.

By computing the standard deviations of depth errors on each *X_W_* coordinate, we know that the standard deviations are small; that is, the depth errors of the corresponding *X_W_* coordinates are closed to their mean. Therefore, the depth errors can be assumed to be the average depth error. Based on abovementioned ideas, the average depth measurement errors listed in Table 3 are plotted in Figure 14. Since the distribution of the average depth measurement errors is approximated linearly, the linear regression method is used to model the depth measurement error. The MATLAB curve fitting toolbox is used to compute the depth error model coefficients and the fitting residuals. Figure 14 shows the average depth measurement error and its approximated linear model. Equation (25) is the linear depth error model derived from MATLAB. Note that *Z_e_* indicates the average depth measurement error.

(25) $$Z_{e}=-0.1635\cdot X+2.996$$

Table 4 shows that the original average depth errors of each corresponding *X_W_* are reduced to below 1.65 mm after the error correction using the linear error model and the greatest error is reduced to 3.45 mm.

## 7. Trajectory Measurement Experiment

**Figure 15.**
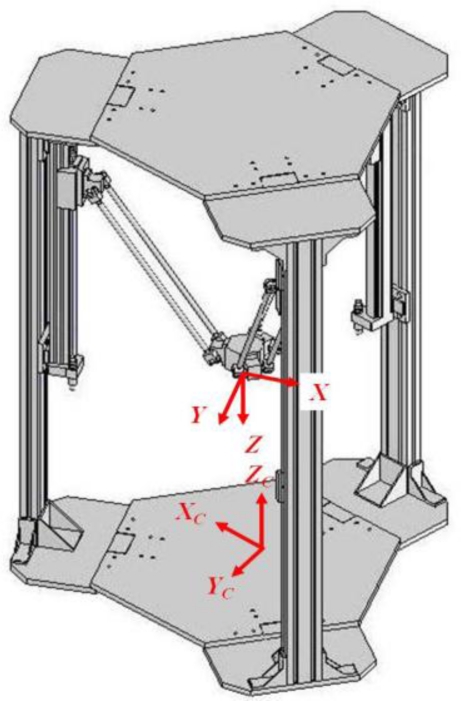
The camera frame and the end-effector frame.

Figure 15 shows the frame assignment of the stereo vision system where {*X_C_*, *Y_C_*, *Z_C_*} is the camera frame and {*X*, *Y*, *Z*} is the end-effector frame. Since the measurement result of stereo vision system is with respect to the camera frame, it needs to be transformed to be with respect to the end-effector frame. The pose relationship between each frame is defined as a homogeneous transformation matrix as Equation (26) shown, where ${\left[\begin{array}{ccc} t_{x} & t_{y} & t_{z} \end{array}\right]}^{T}$ is the origin of the end-effector relative to the camera frame. Note that all the experiment results shown in the following sections are transformed using Equation (26) to be in the consistent coordinates with the end-effector frame:

(26) $$\left[\begin{array}{c} X\\ Y\\ Z\\ 1 \end{array}\right]=\left[\begin{array}{cccc} -\frac{1}{2} & -\frac{\sqrt{3}}{2} & 0 & -t_{x}\\ \frac{\sqrt{3}}{2} & -\frac{1}{2} & 0 & -t_{y}\\ 0 & 0 & 1 & -t_{z}\\ 0 & 0 & 0 & 1 \end{array}\right]\cdot \left[\begin{array}{c} X_{C}\\ Y_{C}\\ Z_{C}\\ 1 \end{array}\right]$$

### 7.1. Fifth Order Polynomial Trajectory

**Figure 16.**
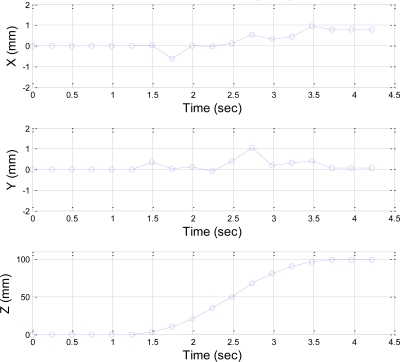
Stereo vision measuring results of a fifth order trajectory with stroke of 100 mm in the X-, Y- and Z-axis.

The desired end-effector trajectories in the Z-direction are given as a fifth order polynomial trajectory with stroke 100 mm in 3 seconds and a fifth order polynomial trajectory with stroke 200 mm in 5 s, respectively. Figure 16 shows the stereo vision measuring results of the fifth order polynomial trajectory with stroke 100 mm.

**Figure 17.**
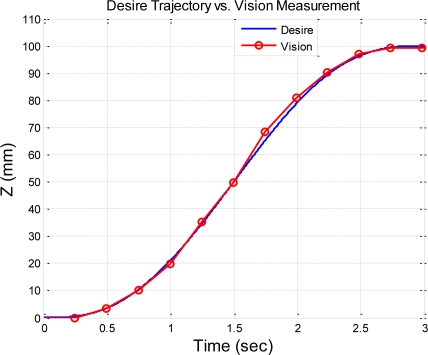
Stereo vision measuring results of a fifth order trajectory with stroke of 100 mm.

The stereo vision measuring results of *X* and *Y* coordinates at both strokes of fifth order trajectory are less than ±2 mm, and the stereo vision measuring results in the *Z*-coordinates show that the end-effector can be positioned to the desired stroke profile. Figure 17 shows the comparison of the desired trajectory and stereo vision measurement results. The end-effector can follow the given desired trajectory well.

### 7.2. Sinusoidal Trajectory

**Figure 18.**
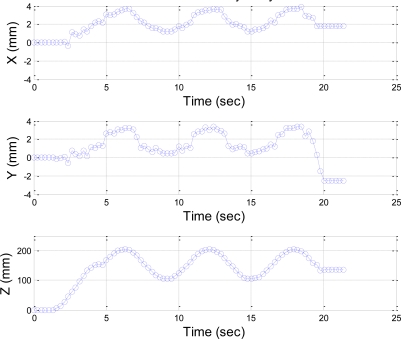
Stereo vision measuring results of sinusoidal trajectory.

**Figure 19.**
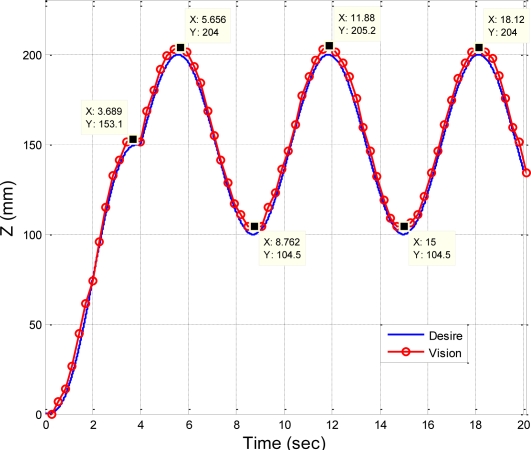
Comparison of the desired sinusoidal trajectory and the stereo vision measuring results.

The desired trajectory of the end-effector in *Z*-direction in this section is planned to be a fifth order polynomial trajectory with stroke 150 mm at t ≤ 4 s, and a sinusoidal trajectory at 4 s ≤ t ≤ 20 s, with amplitude of 50 mm and frequency of 1 rad/s. Figure 18 shows the stereo vision measuring results of the sinusoidal trajectory, and Figure 19 shows the comparison of the desired trajectory and the stereo measurement results. The measurement results of *X* and *Y* coordinates of sinusoidal trajectory are less than ±4 mm, and the difference between the desired sinusoidal trajectory and the measuring results is approximately ±4 mm at the peak of sinusoidal trajectory, which results from the effect of systematic error of the stereo vision system or the vibration of the end-effector during trajectory tracking.

## 8. Conclusions

This paper proposes a stereo vision 3D position measurement system for a three-axial pneumatic parallel mechanism robot arm. The stereo vision 3D position measurement system serves to measure the 3D trajectories of the end-effector of the robot arm. To track the end-effector of the robot arm, the circle detection algorithm is used to detect the desired target and the SAD algorithm is used to track the moving target and to search the corresponding target location along the conjugate epipolar line in the stereo pair. After camera calibration, both intrinsic and extrinsic parameters of the stereo rig can be obtained, so images can be rectified according to camera parameters. Through the use of the epipolar rectification, the stereo matching process is reduced to a horizontal search along the conjugate epipolar line. Finally, 3D trajectories of the end-effector were computed by the stereo triangulation.

In the experiments of this paper, the stereo calibration results, the image rectification results, the circle detection results and the stereo tracking results were shown graphically. In the practical stereo vision measurement experiments, the measuring error of *Z* direction has been corrected first, and the corrected measurement results show that the maximum average error of *Z* direction can be reduced to 2.18 mm. After correcting the measurement error, the end-effector of the three-axial pneumatic parallel mechanism robot arm was planned to track the fifth order polynomial trajectory and the sinusoidal trajectory. These trajectories were then successfully measured and tracked by the stereo vision 3D position measurement system developed in this paper. Future work on the stereo vision 3D position measurement system proposed in this paper can be suggested. To broaden the field of view, a fisheye lens can be used. Using a position sensor such as a laser range finder to calibrate stereo vision system requires the sensor fusion data of laser and stereo vision, and should achieve more reliable and more accurate measurements.

## Figures and Tables

**Table 1.** The specifications of the CCD Camera and image acquisition card.

**Item**	**Specification**	
CS8550Di CCD Camera	Image sensor	All Pixel’s Data Read-out Interline CCD
	Video output pixels	648(H) × 492(V) (Under non-interlace)
	Scanning area	1/3 ”
	Scanning lines	525 lines
	Interlace	1/60 s Non-interlace mode 1/120 s 2:1 Interlace mode
	S/N	52 dB(p-p)/rms
	Power source	DC12 V ± 10%
	Power consumption	Approx. 1.8 W
	Lens mount	C-MOUNT

IMAQ PCI-1410	Available Formats	RS-170/NTSC 30 frames/s interlaced CCIR/PAL 25 frames/s interlaced VGA 60 Hz, 640 × 480 resolution
	Interface	PCI
	Channel	4
	Resolution	8 or 10 bits
	Onboard Memory	16 MB SDRAM
	Sampling Rate	2 M∼40 MHz
	S/N	56 dB

**Table 2.** The intrinsic and extrinsic parameters of the stereo rig.

**Left Camera**			**Right Camera**		
Focal Length	Horizontal	*α* = 848.80271 (pixel)	Focal Length	Horizontal	*α* = 850.84235 (pixel)
	Vertical	*β* = 848.42222 (pixel)		Vertical	*β* = 851.00526 (pixel)
Skew	*γ* = 0		Skew	*γ* = 0	
Principle Point	*u*_0_ = 323.29556 (pixel) *v*_0_ = 231.51767 (pixel)		Principle Point	*u*_0_ = 320.44027 (pixel) *v*_0_ = 254.04592 (pixel)	
Distortion (Radial)	*κ*_1_ = −0.36358 *κ*_2_ = 0.14213		Distortion (Radial)	*κ*_1_ = −0.23851 *κ*_2_ = 0.04721	
Extrinsic Parameters	Rotation		$R=\left[\begin{array}{ccc} 1.0000 & 0.0008 & -0.0046\\ -0.0008 & 1 & 0.0018\\ 0.0046 & -0.0018 & 1 \end{array}\right]$		
	Translation		$T=\left[\begin{array}{ccc} -76.53583 & 0.18358 & 0.14324 \end{array}\right]$ (mm)		

**Table 3.** Depth (*Z_W_*) measurement error at different *X_W_* coordinates (unit: mm).

*X_W_*	−80	−60	−40	−20	0	20	40	60	80	100	120
*Z_W_*											
415					1.93	−1.02	−3.88	−6.68	−9.41		
435					2.23	−0.07	−4.52	−5.59	−9.87		
455				6.06	2.02	−0.64	−3.21	−5.73	−9.44	−13.08	
475				4.83	1.77	−1.23	−4.15	−5.65	−9.87	−12.65	
495			9.69	6.05	2.52	−0.88	−4.17	−5.77	−9.11	−12.24	
515			10.31	4.38	2.32	0.47	−3.31	−9.06	−10.68	−12.16	−15.87
535			9.24	4.14	1.59	−2.39	−4.29	−8.13	−9.98	−12.46	−16.24
555		12.01	7.12	4.25	1.73	−0.91	−7.42	−8.25	−9.98	−13.94	−18.14
575		12.84	9.97	4.55	1.84	−0.73	−3.27	−5.74	−10.77	−13.15	−15.67
595	19.14	12.93	9.8	6.69	3.84	−1.8	−4.54	−7.34	−10.08	−12.68	−15.37
615	18.63	15.22	8.37	5.18	2.1	−1.1	−4.12	−7.15	−10.14	−13.01	−15.91
635	16.18	12.45	8.85	4.58	1.81	−1.61	−5.04	−4.97	−8.81	−11.43	−14.53
S.D.	1.58	1.25	1.04	0.89	0.59	0.75	1.12	1.30	0.58	0.68	1.10
Avg. Error	17.98	13.09	9.17	5.07	2.14	−0.99	−4.33	−6.67	−9.85	−12.68	−15.96

**Table 4.** Depth (*Z_W_*) measurement error after correction (unit: mm).

*X_W_*	−80	−60	−40	−20	0	20	40	60	80	100	120
*Z_W_*											
415					1.95	−0.68	0.74	1.11			
435					0.48	0.33	0.33	0.14	0.14		
455				1.25	−0.74	1.21	0.68	1.22	0.55		
475				0.39	−0.3	−0.51	−0.13	0.02	0.31	0.62	
495			0.72	0.38	0.25	1.8	1.8	1.61	1.56	1.42	
515			1.57	0.75	0.36	−0.14	1.27	2.54	2.12	1.57	
535			0.71	1.82	0.94	−0.05	1.19	2.27	3.35	2.34	1.51
555		1.34	−0.31	0.5	1.52	0.05	0.98	1.87	0.36	1.34	−0.11
575		2.26	0.48	1.16	1.68	2.43	0.53	1.17	1.77	2.46	0.62
595	−0.16	0.26	0.45	0.21	−1.56	1.72	1.94	−0.29	0.05	3.14	3.45
615	−0.78	−0.72	−0.73	2.52	−0.55	−0.32	−0.26	3.00	0.06	0.15	0.39
635	0.09	−0.13	−0.41	2.13	−0.79	−0.82	−1.08	1.78	1.44	1.83	1.78
Avg. Error	−0.28	0.60	0.31	1.11	0.27	0.42	0.67	1.37	1.06	1.65	1.27
